# An In Vitro Study on Mitochondrial Compensatory Response Induced by Gliadin Peptides in Caco-2 Cells

**DOI:** 10.3390/ijms20081862

**Published:** 2019-04-15

**Authors:** Antonella Orlando, Guglielmina Chimienti, Vito Pesce, Flavio Fracasso, Angela Maria Serena Lezza, Francesco Russo

**Affiliations:** 1 Laboratory of Nutritional Pathophysiology, National Institute of Gastroenterology “S. de Bellis”, Research Hospital, 70013 Castellana Grotte (Bari), Italy; antonella.orlando@irccsdebellis.it; 2 Department of Biosciences, Biotechnologies and Biopharmaceutics, University of Bari Aldo Moro, Via Orabona 4, 70100 Bari, Italy; guglielminaalessandra.chimienti@uniba.it (G.C.); vito.pesce@uniba.it (V.P.); flavio.fracasso@uniba.it (F.F.); angelamariaserena.lezza@uniba.it (A.M.S.L)

**Keywords:** Caco-2 cells, gliadin, gluten-related disorders, mitochondrial biogenesis, mtDNA, mtDNA damage, oxidative stress, PGC-1α, PrxIII

## Abstract

Dietary gliadin may show a broad spectrum of toxicity. The interplay between mitochondria and gliadin-induced oxidative stress has not been thoroughly examined in the intestinal epithelium. In this kinetic study, Caco-2 cells were exposed for 24 h to pepsin-trypsin-digested gliadin, alone or in combination with the antioxidant 2,6-di-tbutyl-p-cresol (BHT), and the effects on mitochondrial biogenesis and mtDNA were studied. Cells ability to recover from stress was determined after 24 h and 48 h of incubation in the culture medium. Gliadin-induced oxidative stress evoked a compensatory response. The stressor triggered a rapid and significant increase of Peroxisome proliferator-activated receptor γ coactivator-1alpha (PGC-1α) and Peroxiredoxin III (PrxIII) proteins, and mtDNA amount. As for the effects of gliadin on mtDNA integrity, strand breaks, abasic sites, and modified bases were analyzed in three mtDNA regions. D-loop appeared a more fragile target than Ori-L and ND1/ND2. The temporal trend of the damage at D-loop paralleled that of the amount of mtDNA. Overall, a trend toward control values was shown 48 h after gliadin exposure. Finally, BHT was able to counteract the effects of gliadin. Results from this study highlighted the effects of gliadin-induced oxidative stress on mitochondria, providing valuable evidence that might improve the knowledge of the pathophysiology of gluten-related disorders.

## 1. Introduction

The complex of proteins called gluten contained in wheat gliadin, together with the homologous proteins from barley and rye, are responsible for a group of human diseases defined as “gluten-related disorders” (namely, autoimmune celiac disease (CD), allergy to wheat, and non-celiac gluten sensitivity (NCGS)) [1]. Genetically predisposed individuals after the ingestion of these proteins develop CD, an inflammatory condition of the small intestine triggered by fragments of gliadin that cross the intestinal epithelium and are presented by antigen-presenting cells to the HLA DQ2/DQ8 T lymphocytes present in the intestinal mucosa [2]. Immuno-toxic gliadin peptides can start both adaptive and innate immune responses ultimately leading to barrier disruption [3] and mucosal damage [4]. Indeed, gliadin shows a broad spectrum of toxicity, including the induction of oxidative stress [5]. The inflammatory response also relies on the “respiratory burst” driven by activated leukocytes that leads to the release of high quantities of reactive oxygen species (ROS) [6]. ROS are abundant in the gastrointestinal (GI) tract, and many GI mucosal pathologies are associated with “blazed ROS production” [7,8]. As for CD, gliadin might affect the pro-oxidant-antioxidant balance in the intestinal mucosa, far beyond the host’s capacity to quench [9,10]. Mitochondria, the alpha and omega of the major cell amount of ROS, play a crucial role in oxidative stress [11]. Mitochondrial abnormalities have been demonstrated in several intestinal inflammatory diseases, and mitochondria-targeted antioxidant treatment strategies have been proposed [12,13,14]. The molecular mechanism linking gluten toxicity with mitochondria, ROS, and inflammation could rely on the ability of mitochondrial ROS to activate the multi-protein caspase-1 activating complex (NLRP3 inflammasome) [15,16], as confirmed in peripheral blood mononuclear cells of celiac patients exposed to gliadin [17].

The recently demonstrated findings of an increased mitochondrial DNA (mtDNA) content in the lymphocytes of patients with CD compared to healthy controls suggest the induction of mitochondrial biogenesis as a compensatory response in CD patient’s cells when harmed by the oxidative stress associated with the disease [12]. The interplay between mitochondria and the gliadin-induced oxidative stress related to the inflammatory response has not been thoroughly examined in the intestinal epithelium. To investigate gliadin toxicity with oxidative imbalance and mitochondria dysfunction in CD, we performed a kinetic study using the human intestinal epithelial Caco-2 cell line as a sufficiently appropriate tool to study in vitro the disease [18]. Cells were exposed to pepsin-trypsin-digested gliadin (PTG), alone or in combination with the synthetic phenolic compound butylated hydroxytoluene (2,6-di-tbutyl-p-cresol (BHT)), which exerts its antioxidant activity as ROS scavenger [19]. In particular, we studied the possible induction of mitochondrial biogenesis and the genotoxic effect on mtDNA after 24 h of exposure to treatments. Additionally, we repeated the determinations after further 24 and 48 h of incubation in culture medium to evaluate the ability of cells to recover from gliadin-induced stress.

## 2. Results

### 2.1. MTT Test

**Figure 1:**
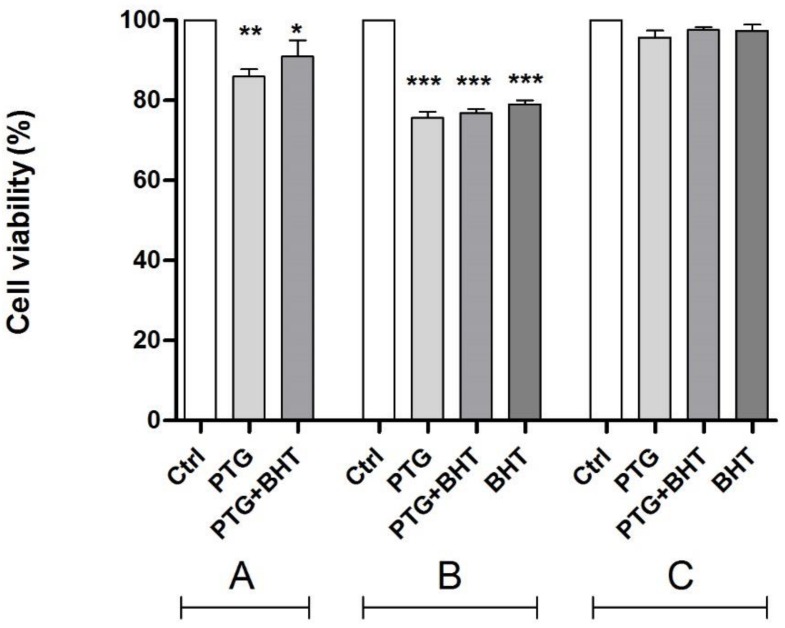
Cell viability in Caco-2 cells by MTT test. Ctrl: untreated control cells; PTG: cells incubated with PTG (1 mg/mL) for 24 h; PTG+BHT: cells co-incubated with PTG (1 mg/mL) and BHT (50 µM) for 24 h; BHT: cells incubated with PTG (1 mg/mL) for 24 h, washed, and incubated with BHT (50 µM) for 24 h. Group **A**: cells harvested at the end of the 24 h treatment. Group **B**: cells washed at the end of the 24 h treatment and further incubated in culture medium for 24 h. Group **C**: cells washed at the end of the 24 h treatment and further incubated in culture medium for 48 h before harvesting. Bars represent the mean and SEM value of three independent experiments conducted in duplicate. Statistical difference determined by One-way ANOVA with Dunnett’s post-test. *: *p* < 0.05; **: *p* < 0.01; ***: *p* < 0.0001. Dunnett’s post-test vs. Ctrl.

Cell viability in treated vs. untreated control cells was evaluated by the MTT test (Figure 1), which relies on the activities of the mitochondrial dehydrogenases. Cells harvested after exposure to PTG for 24 h showed a reduction of the activities of dehydrogenases resulting in a significant (*p* < 0.01) reduction of the percentage of viable cells (−14.1 %) compared to untreated control cells. When BHT was co-administered with PTG, the ROS scavenger was only partially able to improve the cellular viability that resulted significantly (*p* < 0.05) reduced by 9.0% in comparison with control cells (group A). The reduction of viability remained significant also in cells subjected to the initial treatment and harvested after 24 h of incubation in the culture medium, with significant 24.4% and 23.2% reductions in PTG cells and in PTG + BHT cells, respectively (*p* < 0.0001). Finally, also in cells exposed to BHT for 24 h after the treatment with PTG, there was a significant (*p* < 0.0001) reduction in the cellular viability equal to 21.0% (group B). No significant differences between treated and control cells were observed in cells harvested 48 h after the initial treatments (group C).

### 2.2. Mitochondrial Biogenesis

The possible effects of mitochondrial biogenesis were evaluated through the determination of the relative amount of the transcription coactivator Peroxisome proliferator-activated receptor γ coactivator-1alpha (PGC-1α) protein, a master regulator of mitochondrial biogenesis, and the relative mtDNA content in treated and untreated control cells.

**Figure 2:**
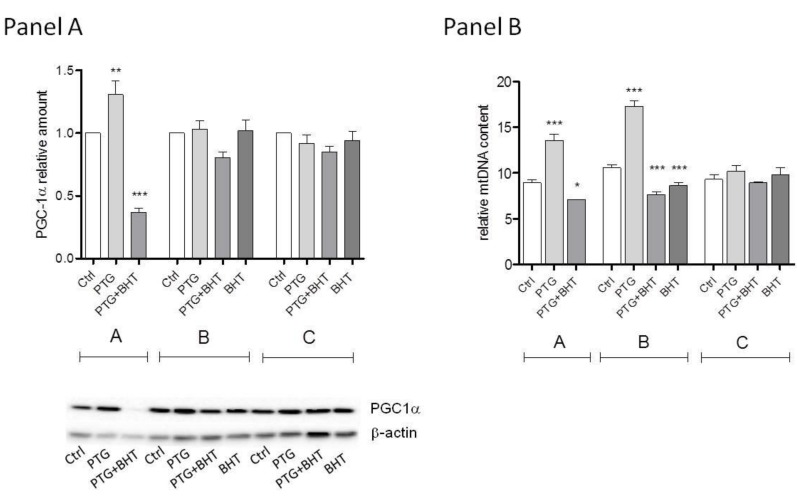
PCG-1α and mtDNA relative contents. Ctrl: untreated control cells; PTG: cells incubated with PTG (1 mg/mL) for 24 h; PTG+BHT: cells co-incubated with PTG (1 mg/mL) and BHT (50 µM) for 24 h; BHT: cells incubated with PTG (1 mg/mL) for 24 h, washed, and incubated with BHT (50 µM) for 24 h. Group **A**: cells harvested at the end of the 24 h treatment. Group **B**: cells washed at the end of the 24 h treatment and further incubated in culture medium for 24 h. Group **C**: cells washed at the end of the 24 h treatment and further incubated in culture medium for 48 h before harvesting. Bars represent the mean and SEM value of at least three independent experiments conducted in duplicate (Panel A) or five independent experiments conducted in triplicate (Panel B). A representative western blot is shown in Panel A. Statistical difference determined by One-way ANOVA with Dunnett’s post-test. *: *p* < 0.05; **: *p* < 0.01; ***: *p* < 0.0001 Dunnett’s post-test vs. Ctrl.

A statistically significant difference in the relative amount of PGC-1α was evident between treated and untreated control cells belonging to group A. A 31% increased amount of protein was observed in PTG cells compared to control ones (*p* < 0.05). When cells were co-exposed to PTG and the synthetic antioxidant BHT a 63% reduction of PGC-1α (*p* < 0.0001) was observed. After further 24 h incubation in culture medium (group B) the difference between treated and untreated cells was still statistically significant (P: 0.0394, One-way ANOVA), but none of the experimental conditions compared to control reached the statistical significance at the post-test. Lastly, no significant differences were observed between cells in group C (Figure 2, Panel A).

As to mtDNA relative content, in group A cells, the mtDNA content was significantly different in the treated and untreated control cells. PTG exposure induced a 60% increase in the mtDNA content relative to untreated cells that was statistically significant at the Dunnett’s post-test (*p* < 0.0001). The ROS scavenger BHT suppressed this increase: an 11% reduction in the mtDNA content was observed in cells simultaneously exposed to PTG and BHT (*p* < 0.05). In cells harvested 24 h after the initial exposure (group B), mtDNA levels were still significantly different among the differently treated cells and controls. In PTG cells, mtDNA appeared to be increased considerably (72% higher) compared to control cells (*p* < 0.0001). The mtDNA content in PTG+BHT cells was significantly reduced by 28% in comparison to control cells (*p* < 0.0001). Interestingly, when the ROS quencher was administered 24 h after the exposure to PTG (BHT cells), only a 17% reduction of mtDNA content occurred (*p* < 0.0001). Of note, when cells were harvested 48 h after the initial exposure (group C), there were no significant differences in mtDNA content relative to control untreated cells (Figure 2, Panel B).

### 2.3. Mitochondrial Antioxidant Response

**Figure 3:**
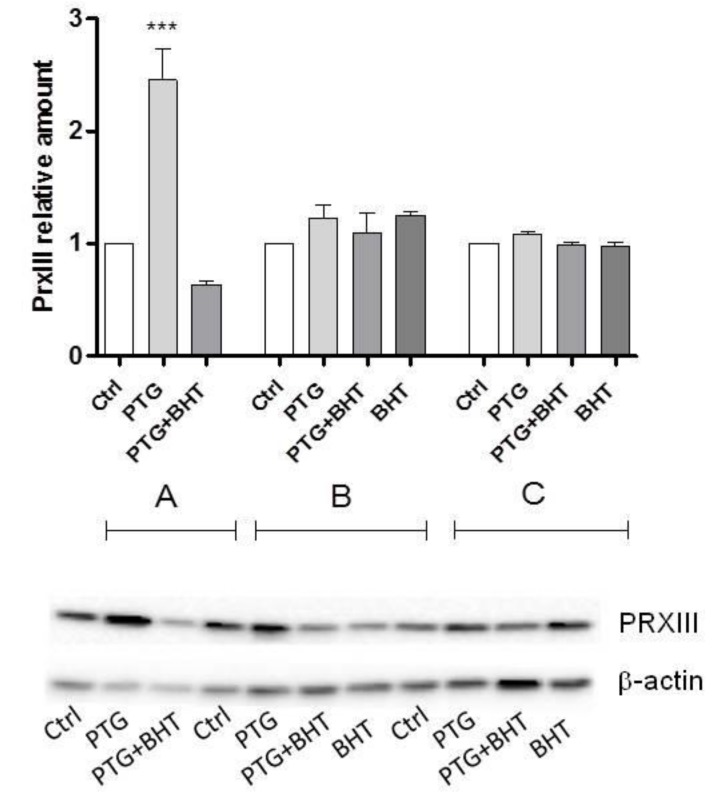
Peroxiredoxin 3 (PrxIII) relative amounts. Ctrl: untreated control cells; PTG: cells incubated with PTG (1 mg/mL) for 24 h; PTG+BHT: cells co-incubated with PTG (1 mg/mL) and BHT (50 µM) for 24 h; BHT: cells incubated with PTG (1 mg/mL) for 24 h, washed, and incubated with BHT (50 µM) for 24 h. Group **A**: cells harvested at the end of the 24 h treatment. Group **B**: cells washed at the end of the 24 h treatment and further incubated in culture medium for 24 h. Group **C**: cells washed at the end of the 24 h treatment and further incubated in culture medium for 48 h before harvesting. Bars represent the mean and SEM value of at least three independent experiments conducted in duplicate. A representative western blot is shown. Statistical difference determined by One-way ANOVA with Dunnett’s post-test. ***: *p* < 0.0001 Dunnett’s post-test vs. Ctrl.

To evaluate whether the exposure to PTG was able to activate cellular antioxidant defenses, the amount of the mitochondrial ROS scavenger protein peroxiredoxin 3 (PrxIII) was determined. Caco-2 cells exposed to the 24 h treatment with PTG showed to be able to orchestrate a statistically significant compensatory antioxidant mechanism as demonstrated by the 2.5-fold increased amount of the mitochondrial enzyme compared to control cells (*p* < 0.0001). In co-treated PTG+BHT cells a 37.0% reduction of PrxIII was found (group A). No significant differences between treated and untreated control cells were evident in groups B and C (Figure 3).

### 2.4. MtDNA Damage

**Figure 4:**
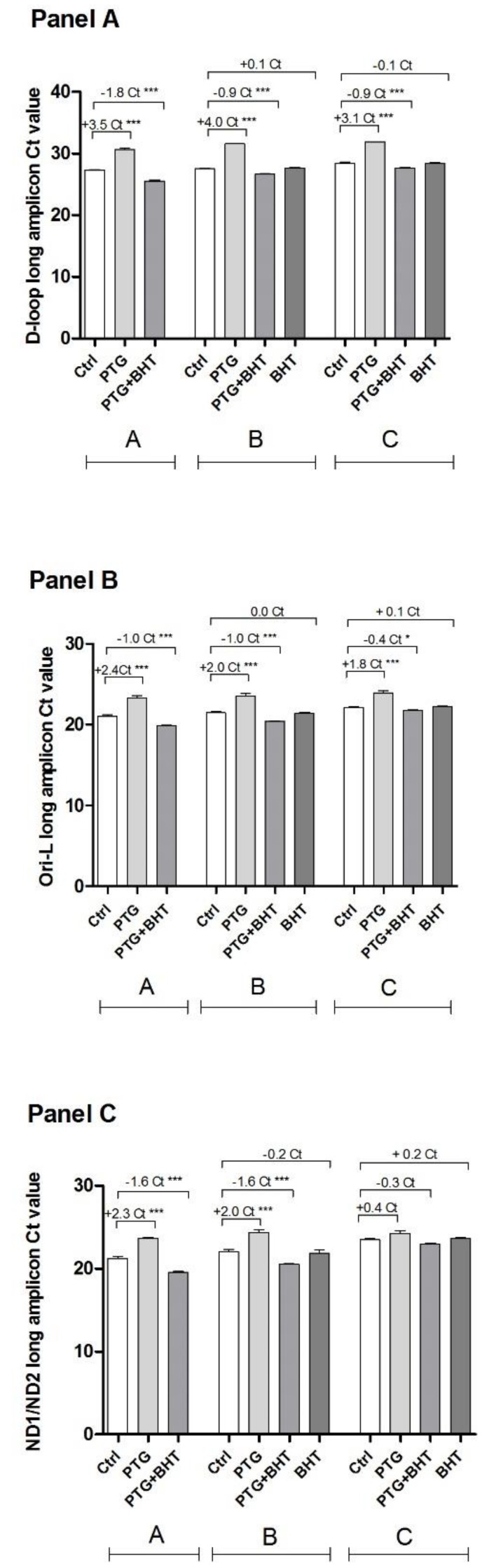
Detection of mtDNA damage. Ctrl: untreated control cells; PTG: cells incubated with PTG (1 mg/mL) for 24 h; PTG+BHT: cells co-incubated with PTG (1 mg/mL) and BHT (50 µM) for 24 h; BHT: cells incubated with PTG (1 mg/mL) for 24 h, washed, and incubated with BHT (50 µM) for 24 h. Group **A**: cells harvested at the end of the 24 h treatment. Group **B**: cells washed at the end of the 24 h treatment and further incubated in culture medium for 24 h. Group **C**: cells washed at the end of the 24 h treatment and further incubated in culture medium for 48 h before harvesting. Bars represent the mean and SEM value of five independent experiments conducted in triplicate. Statistical difference determined by One-way ANOVA with Dunnett’s post-test. *: *p* < 0.05; ***: *p* < 0.0001 Dunnett’s post-test vs. Ctrl.

The investigation of a genotoxic effect induced by gliadin on mtDNA, along with the search for possible hot spot regions was performed by a semi-long qPCR approach. Two regulatory (D-loop and Ori-L) and one coding (ND1/ND2) regions along the mtDNA molecule were analyzed. When the long amplicon encompassing the D-loop region was amplified, a statistically significant difference was found between the value of the Ct of treated and untreated control cells belonging to group A. The Ct of cells treated with PTG was 3.5 cycles higher than that of the untreated control cells (*p* < 0.0001). According to the 2^ΔCt^ formula, this difference corresponds to 11-fold greater damage in PTG cells compared to control ones. As to the effect of the ROS scavenger, cells co-incubated with BHT showed a significant decrease in the threshold Ct cycle compared with the untreated control cells (*p* < 0.0001), consequently the ΔCt was a negative number (−1.8) corresponding to 0.3-fold lower damage than untreated cells. When group B cells were considered the difference in Ct values was significant. The Ct value of PTG cells was 4.0 cycles higher than the Ct of the corresponding untreated control cells (16-fold greater damage) (*p* < 0.0001). The ΔCt of PTG+BHT cells was −0.9 (*p* < 0.0001), indicative of 0.5-fold lower damage compared to control cells. When cells exposed to BHT after PTG were considered in comparison with control ones, no significant difference was present in the ΔCt. Ct values of cells harvested 48 h after treatment were still significantly different (group C). Three-point-one ΔCt (representing an 8-fold greater damage) between PTG and control cells was found (*p* < 0.0001) whereas −0.9 ΔCt (0.5-fold lower damage) was found between PTG+BHT cells and control ones (*p* < 0.0001). Finally, no significant difference was found in the Ct values of cells exposed to BHT after PTG and control cells (Figure 4, Panel A).

As to Ct of the long amplicons of the Ori-L region, the ΔCt between treated and untreated control cells harvested at the end of a 24 h incubation was statistically significant (group A). The Dunnett’s post-test revealed the significance of the ΔCts (*p* < 0.0001) between control cells and both PTG (2.4 ΔCt) and PTG+BHT (−1.0 ΔCt) cells. These variations of Ct value correspond to 5.3-fold increased damage in PTG cells and 0.5-fold decreased damage in PTG+BHT cells compared to controls, respectively. When cells harvested 24 h after the initial treatments along with those treated with BHT after PTG were considered (group B), the difference of Ct values was significant. PTG cells showed statistically significant +2.0 Ct value increase (corresponding to 4-fold increased damage) compared to controls (*p* < 0.0001), PTG+BHT cells showed a statistically significant −1.0 ΔCt (2-fold reduction of damage) (*p* < 0.0001), whereas the difference between BHT cells and controls was not statistically significant. Looking at group C cells, the values of Ct were significantly different. At the post-test, the ΔCt between PTG and control cells showed to be significantly increased (*p* < 0.0001) (1.8 ΔCt, corresponding to approximately 4-fold increased damage). Otherwise, the ΔCt between PTG+BHT and control cells resulted significantly (*p* < 0.05) decreased (−0.4 ΔCt, equal to 0.8-fold reduced damage). The ΔCt between BHT and control cells did not reach the significance (Figure 4, Panel B).

When the ND1/ND2 region was analyzed, the difference of Ct between treated and untreated control cells was statistically significant in both group A and group B cells. The ΔCts between PTG and control cells were +2.3 and +2.0 in the first and the latter group of cells, respectively. These ΔCts were statistically significant (*p* < 0.0001) and corresponded to a 4-fold increase in the damage. As to PTG+BHT cells, the difference of Ct values in comparison with control cells was −1.6 (0.3-fold decreased damage) (*p* < 0.0001) in both group A and group B cells. The difference between BHT and control cells was not statistically significant in cells belonging to group B. Cells in group C showed a statistically significant difference of Ct values (*p*: 0.03228, One-way ANOVA) as concerns the different treatments. When the post-test was applied, no significant differences between treated and control cells resulted (Figure 4, Panel C).

Short amplicons relative to all the three analyzed regions did not differ more than 1 Ct between compared samples.

### 2.5. Apoptosis

**Figure 5:**
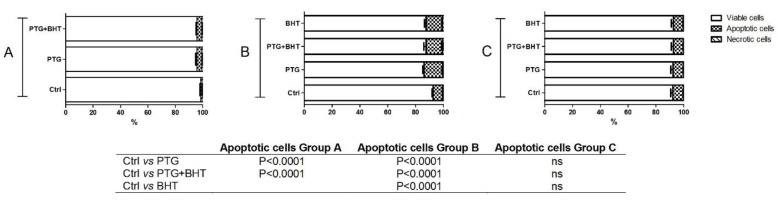
Apoptotic response of Caco-2 cells. Ctrl: untreated control cells; PTG: cells incubated with PTG (1 mg/mL) for 24 h; PTG+BHT: cells co-incubated with PTG (1 mg/mL) and BHT (50 µM) for 24 h; BHT: cells incubated with PTG (1 mg/mL) for 24 h, washed, and incubated with BHT (50 µM) for 24 h. Group **A**: cells harvested at the end of the 24 h treatment. Group **B**: cells washed at the end of the 24 h treatment and further incubated in culture medium for 24 h. Group **C**: cells washed at the end of the 24 h treatment and further incubated in culture medium for 48 h before harvesting. Bars represent the mean and SEM value of three independent experiments conducted in duplicate. Statistical difference determined by One-way ANOVA with Dunnett’s post-test.

To verify whether PTG treatment could exert pro-apoptotic effect and to evaluate the time-course of the event, the percentage of apoptotic cells was assessed in Caco-2 cells subjected to the different experimental conditions. As shown in Figure 5, 24 h of exposure to PTG induced a significant (*p* < 0.0001) increase in the percentage of the apoptotic cells (3.7%) compared to control ones. When cells were co-administered with PTG and BHT for 24 h, the percentage of the apoptotic cells also significantly (*p* < 0.0001) increased (3.6%) (group A). The pro-apoptotic effect persisted and appeared more evident in cells belonging to group B. In PTG cells the apoptotic ones represented 13.5% of the total population, significantly different from the percentage of apoptotic cells in untreated control ones (*p* < 0.0001). In PTG+BHT and BHT cells also, the percentage of apoptotic cells was significantly higher (*p* < 0.0001) compared to that observed in untreated cells (11.9% in both the experimental conditions). Finally, in group C cells, no significant differences were found in the percentage of apoptotic cells in treated vs. untreated control ones (Figure 5).

## 3. Discussion

Oxidative stress plays a role in many GI diseases [20], including chronic inflammatory autoimmune CD [5,9,10,12,21]. Apart from dietary gliadin [9], in vitro studies have assessed the ability of the PTG at a concentration of 1 mg/mL to cause oxidative imbalance [10,22]. Several other authors [23,24,25] have already used 1 mg/mL PTG, proving this concentration effective in unveiling its biological effects and also our group has used in a previous study 1 mg/mL PTG to evaluate the protective role of *Lactobacillus rhamnosus* GG against the alterations of paracellular permeability induced by this protein [3].

Since mitochondria represent not only the origin, through the respiratory chain, but also the target for the major cellular amount of ROS we investigated mitochondrial effects induced by gliadin peptides. In this kinetic study, Caco-2 cells were exposed to PTG alone or in combination with the antioxidant BHT [19] for 24 h. The cellular responses were evaluated immediately after the exposures as well as after further 24 h or 48 h of incubation in culture medium to assess the cellular ability to recover from PTG-induced stress. In addition, to better investigate the complex relationship between ROS and synthetic antioxidants [26], cellular responses were also evaluated in cells exposed to BHT only after the induction with PTG.

PTG exposure was able to affect mitochondrial metabolism, as shown by the here reported reduction of mitochondrial dehydrogenases activities in PTG compared to control cells. The decrease in such activities impacted on cells viability resulting in a mild cytotoxic effect, as already reported by others [10]. The entity of cytotoxicity we have determined through the MTT assay appears to be smaller than that reported in PTG-treated Caco-2 cells by Giovannini et al., which was evaluated by cell counting [27]. An overestimation of viable cells could explain this discrepancy. This finding could be due to an increased dehydrogenase activity per cell caused by the PTG-induced mitochondrial biogenesis as unveiled in the present paper. However, it appears that this cytotoxic effect relied not exclusively on the induced oxidative stress, as demonstrated by the limited capacity of BHT to counteract cytotoxicity. These findings confirm that gliadin-induced enteropathy depends on several pathological mechanisms [1]. The absence of significant differences in cellular viability among treated and untreated cells harvested 48 h after the exposure to PTG is indicative of the ability of these cells to rescue from the mild PTG-induced cytotoxicity.

The observed PTG-mediated reduction in mitochondrial functionality, as demonstrated by the MTT assay results, led us to investigate mitochondrial biogenesis in our experimental model. Mitochondrial biogenesis is a complex process, which involves proteins encoded by both the mitochondrial and nuclear genomes, with PGC-1α as the master regulator of the nucleus-mitochondrion crosstalk [28]. Reactive molecular species such as ROS and reactive nitrogen species (RNS) are now recognized as transducing signals able to regulate transcription factors to adapt to an ever-changing cellular environment [29,30]. The significant increase of PGC-1α amount that was observed in cells exposed to PTG compared to control ones indicates the induction of mitochondrial biogenesis that well fits with a scenario consisting of increased ROS amount induced by the stressor gliadin [10,22]. Besides, the significant reduction of PGC-1α amount in cells co-exposed to PTG and the ROS scavenger BHT points out to the importance of the fine-tuning of the balanced cellular oxidative status. Antioxidants are molecules involved in the scavenging of reactive species causing oxidative stress. However, considering the role of ROS as signaling molecules in intracellular processes [31], the disruption of the redox status by supplementation of exogenous antioxidants might have harmful effects. One can hypothesize that in the co-exposed cells the antioxidant BHT scavenged not only the induced ROS but also the endogenous ones, which are involved in cellular physiological functions [31]. As concerns the time-course of PGC-1α expression, Caco-2 cells showed to be able to soon recover from the opposing effects of both PTG and PTG+BHT.

Oxidative stress, according to its severity, can act differently on mitochondrial biogenesis, as already demonstrated by others on the mtDNA content. In particular, mild oxidative stress exerted by various kinds of stressors has been reported to induce an increase in mtDNA content in different tissues or cell lines. Vice versa, severe oxidative stress led to the loss of mtDNA [32,33,34]. These reports support the biphasic nature of the oxidative stress stimulus as a regulator of mitochondrial biogenesis that was initially proposed by the mitohormesis theory [35] and later consistently demonstrated [12,36].

The molecular setting that appeared in PTG-treated cells consisted of a significant increase in mtDNA content compared to untreated cells. Such an increase reached the zenith 24 h after the exposure and mtDNA content returned to control values after further 24 h. The capacity of the ROS scavenger to suppress this increase provides evidence for a trigger caused by the PTG-induced oxidative stress. The tendency to return to control cell values, with particular reference to the absence of significant difference between PTG and controls in the group C cells, could be indicative of the effectiveness of the cellular antioxidant defense system to restore the redox balance. Notably, an increase in mtDNA content was already reported in the clinical setting of several conditions associated with mild oxidative stress such as the exposure to environmental pollutants [37], aging [38], diabetic retinopathy [39], or CD [12].

The originality of the present study also comes from the simultaneous evaluations of PGC-1α protein expression and mtDNA content that allow the temporal comparison of different steps along the pathway of mitochondrial biogenesis. PTG-induced mild oxidative stress led to an increase in both PGC-1α and mtDNA contents already after the initial exposure. However, while the rise in PGC-1α content was abolished in the following 24 h, that in mtDNA was further raised. This shift demonstrates the temporal succession of events along the pathway, which begun with the induction of the master regulator of mitochondrial biogenesis and longer lasted in its final step of mtDNA replication. Further support of this hypothesis derives from the data obtained in PTG + BHT cells. In fact, a concomitant decrease in PGC-1α and mtDNA was observed in the co-treated cells immediately after treatment. After 24 h the decrease was abolished for PGC-1 α while lasting longer for mtDNA content.

To scavenge the excessive ROS cells are provided with antioxidants and antioxidant enzymes. The main antioxidant enzymes are those in mitochondria, which are the subcellular compartment in which most ROS are generated [40]. PrxIII, a mitochondrial protein with antioxidant function, is among the antioxidant transactivated by PGC-1α [41]. Indeed, a significant increase in the amount of this enzyme was observed in PTG-exposed cells and paralleled that of PGC-1α. The cellular antioxidant response was time limited since a return to control values was soon evident in group B cells.

Other authors have already demonstrated a genotoxic effect of gliadin peptides on total DNA in Caco-2 cells using the comet assay [22]. In the present study, we investigated on mtDNA damage, given that mtDNA is known to be particularly vulnerable to oxidative lesions that in turn can result in dysfunctional mitochondria [42]. MtDNA lesions induced by oxidative stress include single- and double-strand breaks, abasic sites, and oxidized DNA bases, with guanine being the most susceptible to oxidation [43]. Since it has been demonstrated that oxidative stress does not induce uniform damage along the mtDNA molecule [44,45] we also investigated whether PTG-induced damage could conform to this feature. As from data here shown, PTG was genotoxic for mtDNA, and the genotoxicity appeared to rely on the PTG-induced oxidative stress since the ROS quencher BHT was able to counteract it.

MtDNA damage presented itself as a premature event that involved all the three analyzed regions, namely the D-loop, Ori-L, and ND1/ND2 regions; however, the D-loop appeared to be a more fragile target as concerns both the degree of the damage and its persistence. This region showed values of ΔCt compatible with those reported by other authors that used the same methodological approach to evaluate mtDNA damage in human primary skin cells exposed to sub-lethal doses of hydrogen peroxides [46]. In group A cells, the ΔCt between long amplicons from PTG and control cells was higher for D-loop than for both Ori-L and ND1/ND2 regions. This feature is indicative of a higher presence of oxidative lesions able to halt or delay the progression of the polymerase [47,48]. During the following 24 h and 48 h after the initial exposure, the ΔCt between PTG and control cells showed the tendency to a progressive reduction as concerns both the Ori-L and ND1/ND2 regions, indicative of the transient feature of the oxidative lesions that underwent to repair during the time.

On the contrary, at the D-loop region, the ΔCt of cells collected 24 h after the end of the incubation with PTG was further increased compared to the ΔCt of cells harvested at the end of treatment. Only in cells harvested 48 h after PTG treatment, the ΔCt showed the tendency to the reduction. Of note, the temporal trend of the variations of ΔCt at the D-loop paralleled that of the relative amount of mtDNA. As from the literature, a “controlled oxidative damage and repair” at the D-loop could explain the enhanced mitochondrial biogenesis observed after the exposure to a mild oxidative stress [45], since the presence of oxidized bases enhances the DNA affinity for the mitochondrial transcription factor A (TFAM) [49], deeply involved in mtDNA transcription and initiation of replication [50].

Finally, results from this study confirm that gliadin induced pro-apoptotic processes in Caco-2 cells [22] and show the cellular ability to rescue from them.

At the best of our knowledge, the present study is the first one highlighting the effects on mitochondria of PTG-induced oxidative stress. The mitochondrial response consisted of a compensatory induction of biogenesis, similar to that evoked in other situations characterized by mild oxidative stress [12,37,38,39]. Evidence from this study further supports the theory of mitohormesis [35], although it does not dissect the molecular mechanisms underlying the response itself.

## 4. Materials and Methods

### 4.1. Cell Culture Conditions

Human colon adenocarcinoma-derived Caco-2 cell line was obtained from the Interlab Cell Line Collection (IST, Genoa, Italy) and routinely cultured in RPMI-1640, 10% fetal bovine serum (FBS), 2 mM glutamine, 100 U/mL penicillin, 100 μg/mL streptomycin, in monolayer culture at 37 °C in 5% CO_2_. All reagents were from Sigma Aldrich (Milan, Italy).

### 4.2. Gliadin Digest

PTG was freshly prepared as previously described by Drago et al. [51] and added to Caco-2 cells at a concentration of 1 mg/mL in PBS.

### 4.3. Cell Treatment

The experimental design of this study consisted of an initial exposure of CaCo-2 cells to PTG alone (1 mg/mL) or in a co-administration with BHT (50 µM) for 24 h (PTG and PTG+BHT, respectively). Cells were immediately harvested (group A) or washed and further incubated in culture medium for 24 h (group B) or 48 h (group C) before harvesting. To highlight the reparative effect by BHT on PTG-induced oxidative damage, in a subset of experiments, cells treated with PTG alone for 24 h, were washed, incubated with BHT (50 µM) for 24 h, and immediately harvested (group B) or washed and further incubated in the culture medium for 24 h (group C) (BHT). Each treatment included its control (untreated cells).

### 4.4. MTT Test

Cell viability has been evaluated by the 3-(4,5di-methylthiazol-2-yl)-2,5-diphenyltetrazolium bromide MTT test. The assay is based on the assumption that the MTT tetrazolium salt reduction is due to the activities of mitochondrial dehydrogenases [52]. After the 24 h exposure or at the end of the following incubation periods in the culture medium, MTT stock solution (5 mg/mL in medium) was added to each dish at a volume of one-tenth the original culture volume and incubated for 2 h at 37 °C in humidified CO_2_. After then, the medium was replaced with acidic isopropanol (0.1 N HCl absolute isopropanol). Formazan formation was spectrophotometrically monitored by at 570 nm.

### 4.5. Western Blots

Protein extracts were obtained treating each pellet from control and treated cells with total lysis buffer (Pierce Ripa buffer, Thermo Scientific, Rockford, IL, USA) supplemented with protease and phosphatase inhibitors (Thermo Scientific, Rockford, IL, USA).

After homogenization and centrifugation at 14,000 rpm for 15 min at 4 °C, protein concentration was measured by a standard Bradford assay (Bio-Rad, Milan, Italy). Aliquots of 10 µg of total protein extracts from each sample were denatured in 5× Laemmli sample buffer and loaded into 4–12% pre-cast polyacrylamide gels (Bio-Rad, Milan, Italy) for western blot analysis. Anti-PGC-1α (NBP1 04676, Ab NOVUS, Centennial, CO, USA) (1:5000), anti-PrxIII (LFPA0030, Ab FRONTIER, Seoul, Korea) (1:5000), and anti-β-actin (A2066, Sigma Aldrich, Milan, Italy) (1:20,000) were used as primary antibodies. After overnight incubation, the membranes were further incubated with a horseradish peroxidase-conjugated rabbit secondary antibody. The proteins were detected by chemiluminescence (Clarity Western ECL substrate, Bio-Rad, Milan, Italy). Signals were analyzed by laser densitometry with the Chemi Doc System and Image Lab software (Bio-Rad Laboratories Inc., Hercules, CA, USA). The densitometric value (OD units) of each band was then normalized against β-actin expression.

### 4.6. mtDNA Content

The relative content of mtDNA was measured by quantitative real-time polymerase chain reaction (qPCR) via SYBR Green chemistry, using 10 ng total DNA as template. The quantification of the mtDNA content (mtDNA primer set) relative to nuclear DNA (β-actin primer set) was determined as previously reported [53]. See Table 1 for primer sequences.

### 4.7. mtDNA Damage Analysis

The mtDNA damage was evaluated searching for the presence of mtDNA strand breaks, oxidatively modified bases, and abasic sites. The applied qPCR assay is based on the halts of the progression of DNA polymerase or the reduction of the efficiency of the amplification due to the presence of lesions on the template, thus resulting in a positive correlation between DNA damage and the number of cycles necessary to reach the Ct [46,47]. Since the sensitivity of the assay depends on the lengths of the amplicon [54], fragments of about 1000 bp encompassing the regulatory regions in mtDNA, namely the D-loop and the origin of replication of L-strand (Ori-L), and one coding region (ND1/ND2 genes) were amplified (semi-long qPCR). The relative abundance of mtDNA lesions in treated relative to untreated control cells was determined according to the 2^ΔCt^ formula, after controlling for any variations in DNA copy number through the simultaneous amplification of short amplicons (range 55–84 bp) from the analyzed mtDNA regions (no more than 1 Ct difference between short products from compared samples). ΔCt is the difference between the Ct values of long amplicons in treated vs. untreated control cells [46]. Reactions were performed via SYBR Green chemistry using 20 ng total DNA as template. After 10 min at 95 °C, amplification proceeded for 40 cycles (95 °C for 10 s, annealing and extension at 60 °C for 60 s for the long amplicons, and 95 °C for 1 s, annealing and extension at 60 °C for 20 s for the short amplicons). See Table 1 for primer sequences.

### 4.8. Apoptosis

The apoptotic process was evaluated by the Muse Cell Analyzer using the Muse Annexin V/Dead Cell kit (Merck-Millipore, Darmstadt, Germany) following the instructions of the supplier.

### 4.9. Statistical Analysis

Data were analyzed by One-way ANOVA analysis of variance and Dunnett’s multiple comparison test for treated vs. untreated control cells. All data are expressed as mean and SEM of at least three independent experiments. Differences were considered significant at *p* < 0.05. A specific software package was used for the statistical analysis (Stata Statistical Software: Release 9; StataCorp LP, College Station, TX, USA).

## 5. Conclusions

Apart from the role in providing power to cells and affecting cell metabolism (e.g., nutritional sensing, synthesis of metabolic precursors, calcium regulation, maintenance of redox regulation and decision in cell fate) [55], mitochondria are relevant in oxidative stress. In this framework, the understanding of the relationship between oxidative stressors, such as gliadin, and these organelles could improve our knowledge of the pathophysiology of gluten-related disorders.

## Figures and Tables

**Table 1:** Oligonucleotide primer sequences.

Primer Set	Forward Primer	Reverse Primer	(nps)	(nps)
mtDNA	5′ ACGCCATAAAACTCTTCACCAAAG 3′	5′ GGGTTCATAGTAGAAGAGCGATGG 3′	3,458–3,481	3,568–3,545
β-actin	5′ TTGGCAATGAGCGGTTCC 3′	5′ AGCACTGTGTTGGCGTAC 3′	124-141	271-254
ND1/2 long	5′ CCCTTCGCCCTATTCTTCAT 3′	5′ GCGTAGCTGGGTTTGGTTTA 3′	3,961-3,980	4,997-4978
ND1/2 short	5′ CCCTTCGCCCTATTCTTCAT 3′	5′ GGAAGATTGTAGTGGTGAGGGT 3′	3,961-3,980	4,033-4012
Ori-L long	5′ CAGCTAAGCACCCTAATCAACTGG 3′	5′ TGGGAGATTATTCCGAAGCCTG 3′	5,696-5,719	6,670-6,649
Ori-L short	5′ CAGCTAAGCACCCTAATCAACTGG 3′	5′ CTTCAAACCTGCCGGGGCT 3′	5,696-5,719	5,780-5,762
D-loop long	5′ CTGTTCTTTCATGGGGAAGC 3′	5′ AAAGTGCATACCGCCAAAAG 3′	16,021-16,040	424-405
D-loop short	5′ CCCTAACACCAGCCTAACCA 3′	5′ AAAGTGCATACCGCCAAAAG 3′	370-389	424-405

Numbering is according to GenBank™ accession number HQ287896.1 (Homo sapiens mitochondrion, complete genome), except for the β-actin primer set which is according to GenBank™ accession number DQ407611 (Homo sapiens, β-actin mRNA). nps: nucleotide positions.
